# Risk factors associated with bleeding after multi antithrombotic therapy during implantation of cardiac implantable electronic devices

**DOI:** 10.1007/s00380-016-0879-x

**Published:** 2016-07-28

**Authors:** Kohei Ishibashi, Koji Miyamoto, Tsukasa Kamakura, Mitsuru Wada, Ikutaro Nakajima, Yuko Inoue, Hideo Okamura, Takashi Noda, Takeshi Aiba, Shiro Kamakura, Wataru Shimizu, Satoshi Yasuda, Takashi Akasaka, Kengo Kusano

**Affiliations:** 1 Department of Cardiovascular Medicine, National Cerebral and Cardiovascular Center, 5-7-1 Fujishiro-dai, Suita, Osaka 565-8565 Japan; 2 Department of Cardiovascular Medicine, Nippon Medical School, Tokyo, Japan; 3 Department of Cardiovascular Medicine, Wakayama Medical University, Wakayama, Japan

**Keywords:** Cardiac surgery, Electrophysiology, Implanted cardiac defibrillators, Pacemakers

## Abstract

Previous studies showed that continuous anticoagulation or single antiplatelet therapy during implantations of cardiac implantable electronic devices (CIED) was relatively safe. However, the safety of continuous multi antithrombotic therapy (AT) in patients undergoing CIED interventions has not been clearly defined. We sought to evaluate the safety of this therapy during CIED implantations. A total of 300 consecutive patients (mean 69 years old, 171 males) with CIED implantations were enrolled in this study. The patients were divided into 6 groups [No-AT, oral anticoagulant therapy (OAT), single antiplatelet therapy (SAPT), OAT and SAPT, dual antiplatelet therapy (DAPT), triple AT (TAT)], and the perioperative complications were evaluated. Clinically significant pocket hematomas (PH) were defined as PH needing surgical intervention, prolonged hospitalizations, interruption of AT, or blood product transfusions. There were 129, 89, 49, 20, 10, and 3 patients in No-AT, OAT, SAPT, OAT + SAPT, DAPT, and TAT groups, respectively. The occurrence of clinically significant PH and thromboembolism did not differ among 6 groups (*p* = 0.145 and *p* = 0.795, respectively). However, high HAS-BLED score and valvular heart disease (VHD) were associated with clinically significant PH (*p* = 0.014 and *p* = 0.015, respectively). Continuous multi AT may be tolerated, but patients with high HAS-BLED score or VHD would require a careful attention during CIED implantations.

## Introduction

Current guidelines recommend that patients without a high thromboembolic risk should stop antithrombotic therapy (AT) because the implantation of cardiac implantable electronic devices (CIED) with the concomitant use of AT poses an increased risk of perioperative bleeding complications [1], but the interruption of antiplatelet and anticoagulant drugs increases thromboembolic events [2–5]. On the other hand, previous studies showed that continuous anticoagulation during CIED implantations was safe, and a previous report revealed that antiplatelet therapy should not be stopped before low-invasive surgeries because local hematomas are easy to control their bleeding [6–9]. Thus, continuous AT during CIED implantations has been recommended recently. In previous reports, a considerable number of patients with multi (dual or triple) AT were included. However, the safety of continuous multi AT in patients undergoing CIED implantations has not been sufficiently evaluated [10–13]. In this study, we sought to evaluate the safety of continuous multi AT during CIED implantations.

## Materials and methods

### Patient population

This study was a retrospective observational study. The study population consisted of consecutive patients who underwent CIED [pacemaker (PM), implantable cardioverter defibrillator (ICD), cardiac resynchronization therapy (CRT)-pacemaker (CRT-P), or CRT-defibrillator (CRT-D)] implantations without heparin bridging therapy in 2012. All patients who underwent the device surgery continued AT through the procedure. All patients underwent procedures with the standard techniques for a pectoral subfascial pocket formation and transvenous lead placement by way of the subclavian vein using tined or screw-in leads. All right atrial and right ventricular leads were positioned in the right auricular appendage and right ventricular apex, respectively. The left ventricular leads were positioned in the lateral, posterolateral, or anterior cardiac vein. Written informed consent was obtained from each patient in this study to undergo a CIED implantation. The privacy of the patients was protected by the anonymization of all data.

### Study protocol

Three hundred patients were enrolled in this study. Patients were divided into 6 groups as follows; No-AT group: patients without any AT, OAT group: patients with oral anticoagulant therapy (OAT), SAPT group: patients with single antiplatelet therapy (SAPT), OAT + SAPT group: patients with OAT and SAPT, DAPT group: patients with dual antiplatelet therapy (DAPT), and TAT group: patients with triple antithrombotic therapy (TAT). They were evaluated for any perioperative complications (bleeding or thromboembolic events) occurring within 30 days of the surgery. Bleeding events included pocket hematomas (PH), clinically significant PH, cerebral bleeding, gastrointestinal bleeding, and cardiac tamponade. The definition of PH was bleeding not requiring additional intervention but requiring treatment with compress. Clinically significant PH were defined as bleeding requiring surgical intervention, prolongation of hospitalization, interruption of AT, and blood product transfusions. Thromboembolic events included strokes, transient ischemic attacks (TIAs), myocardial infarctions, pulmonary embolisms, and deep vein thrombosis. Furthermore, the predictors of clinically significant PH were evaluated.

### Patient data collection and perioperative risk evaluation

The patient characteristics, including the co-morbidities and medication history, and procedural details were collected at the time of the CIED implantation. The HAS-BLED [hypertension, abnormal renal/liver function, stroke, bleeding history or predisposition, labile international normalized ratio, elderly (>65 years), drugs/alcohol] score was used to assess the bleeding risk, and scored hypertension (HT), abnormal renal/liver function (1 point each), strokes, bleeding history or a predisposition to it, labile international normalized ratio, being elderly (>65 years), and drugs/alcohol use (1 point each) [14]. If the HAS-BLED score was ≥3, a patient was considered to have a considerable risk of bleeding [15–17]. Every thromboembolic risk factor was evaluated and we assessed the thromboembolic risk using the CHADS2 and CHA2DS2-VASc scores [14, 17–19]. The CHADS2 score assigned 1 point each for congestive heart failure (CHF), HT, age ≥75 years, and diabetes mellitus (DM), and 2 points for a history of a stroke or TIA [14]. The CHA2DS2-VASc score assigned 1 point each for CHF, HT, age 65–74 years, DM, vascular disease, a female sex; and 2 points for an age ≥75 years, and a history of a stroke or TIA [18]. If the CHA2DS2-VASc score was ≥2, a patient was considered to have a considerable risk of a thromboembolism [19].

### Statistical analysis

The statistical analyses were performed using JMP version 9 software (SAS Institute Inc., Tokyo, Japan). The results are expressed as the mean ± SD for continuous variables. Categorical data are presented as numbers (%). Differences among groups were analyzed by using the *t-*test for unpaired data, Chi-square test, and Fisher exact test, as appropriate. Differences in continuous variables were assessed using a one-way analysis of variance (ANOVA). A *p* value <0.05 was considered significant. Logistic regression analysis was used to estimate the magnitude of association [i.e., odds ratios (ORs)] between clinically significant PH and clinical characteristics.

## Results

### Clinical characteristics of No-AT, OAT, SAPT, OAT + SAPT, DAPT and TAT groups

The baseline characteristics are listed in Table 1. The number of patients in No-AT, OAT, SAPT, OAT + SAPT, DAPT, and TAT groups was 129, 89, 49, 20, 10 and 3, respectively. The mean patient age was 69 years; 171 (57 %) were male. The frequency of ischemic heart disease, valvular heart disease (VHD), and atrial fibrillation was 18, 8 and 27 %, respectively. The rate of anticoagulant drug use was 37 %, and a novel oral anticoagulant (NOAC) use was observed in only 5 patients in this study. The prothrombin time-international ratio (PT-INR) as a warfarin control parameter was 1.7 and there was no significant difference in the PT-INR among OAT, OAT + SAPT, and TAT groups. The rate of antiplatelet drug use was 32 %.

**Table 1:** Comparison of the clinical characteristics among No-AT, OAT, SAPT, OAT + SAPT, DAPT, and TAT groups

	Total (*n* = 300)	No-AT (*n* = 129)	OAT (*n* = 89)	SAPT (*n* = 49)	OAT + SAPT (*n* = 20)	DAPT (*n* = 10)	TAT (*n* = 3)	*p* value
Age (years)	69 ± 16	66 ± 19	69 ± 14	75 ± 11	72 ± 10	65 ± 12	64 ± 21	0.017
Male	171 (57)	68 (53)	41 (46)	34 (69)	15 (75)	10 (100)	3 (100)	<0.001
Body height (cm)	159 ± 11	159 ± 11	158 ± 10	159 ± 8	162 ± 10	167 ± 10	166 ± 3	0.155
Body weight (kg)	55 ± 11	55 ± 11	53 ± 12	56 ± 10	56 ± 11	62 ± 9	62 ± 6	0.101
BMI	22 ± 3	22 ± 3	21 ± 4	22 ± 3	21 ± 4	22 ± 2	23 ± 3	0.349
Serum creatinine (mg/dl)	1.1 ± 0.7	0.9 ± 0.4	1.2 ± 0.8	1.4 ± 1.1	1.3 ± 0.6	1.0 ± 0.2	1.1 ± 0.3	<0.001
Blood hemoglobin (g/dl)	12.7 ± 1.8	13.0 ± 1.6	12.6 ± 1.8	12.6 ± 1.8	12.5 ± 2.2	12.2 ± 2.4	12.4 ± 1.4	0.439
Diabetes mellitus	68 (23)	16 (12)	19 (21)	18 (37)	9 (45)	5 (50)	1 (33)	<0.001
Hypertension	147 (49)	61 (47)	32 (36)	35 (71)	11 (55)	6 (60)	2 (67)	0.004
Ischemic heart disease	54 (18)	3 (2)	2 (2)	26 (53)	11 (55)	9 (90)	3 (100)	<0.001
Valvular heart disease	23 (8)	2 (2)	18 (20)	1 (2)	2 (10)	0 (0)	0 (0)	<0.001
Atrial fibrillation	82 (27)	11 (9)	52 (58)	2 (4)	15 (75)	0 (0)	2 (67)	<0.001
Drug								
Anticoagulant drug								
Warfarin	107 (36)	–	85 (96)	–	19 (95)	–	3 (100)	–
Control of PT-INR	1.7 ± 0.4	–	1.7 ± 0.4	–	1.8 ± 0.5	–	1.4 ± 0.3	–
NOAC	5 (1)	–	4 (4)	–	1 (5)	–	0 (0)	–
Antiplatelet drug								
Aspirin	74 (25)	–	–	41 (84)	20 (100)	10 (100)	3 (100)	–
Thienopyridine	13 (4)	–	–	2 (4)	0 (0)	8 (80)	3 (100)	–
Cilostazol	8 (3)	–	–	6 (12)	0 (0)	2 (20)	0 (0)	–
β-Blocker	138 (46)	39 (30)	55 (62)	23 (47)	13 (65)	6 (60)	2 (67)	<0.001
ACE inhibitor/ARB	143 (48)	45 (35)	52 (58)	27 (55)	9 (45)	7 (70)	3 (100)	0.002
Statin	101 (34)	27 (21)	25 (28)	29 (59)	10 (50)	7 (70)	3 (100)	<0.001
Diuretics	141 (47)	32 (25)	65 (73)	24 (49)	11 (55)	6 (60)	3 (100)	<0.001
Amiodarone	51 (17)	10 (8)	22 (25)	10 (20)	4 (20)	3 (30)	2 (67)	0.002

Values are given as the *n* (%) or mean ± standard deviation

*ACE* angiotensin converting enzyme, *ARB* angiotensin receptor blocker, *AT* antithrombotic therapy, *BMI* body mass index, *DAPT* dual antiplatelet therapy, *NOAC* novel oral anticoagulant, *OAT* oral anticoagulant therapy, *PT-INR* prothrombin time-international ratio, *SAPT* single antiplatelet therapy, *TAT* triple antithrombotic therapy

### Procedural data of No-AT, OAT, SAPT, OAT + SAPT, DAPT and TAT groups

The frequency of a large device (ICD and CRT-D), de novo implantation, and system upgrade were 44, 63 and 3 %, respectively. The mean procedure time was 1.8 h. There were no significant differences among the 6 groups regarding the CRD type, rate of de novo implantations, system upgrades, or procedure time (Table 2).

**Table 2:** Comparison of the procedural data among No-AT, OAT, SAPT, OAT + SAPT, DAPT, and TAT groups

	Total (n = 300)	No-AT (*n* = 129)	OAT (*n* = 89)	SAPT (*n* = 49)	OAT + SAPT (*n* = 20)	DAPT (*n* = 10)	TAT (*n* = 3)	*p* value
Type of CIED								0.522
PM and CRT-P	167 (56)	75 (58)	52 (58)	27 (55)	8 (40)	4 (40)	1 (33)	
ICD and CRT-D	133 (44)	54 (42)	37 (42)	22 (45)	12 (60)	6 (60)	2 (67)	
De novo implantation	190 (63)	78 (60)	54 (61)	35 (71)	13 (65)	7 (70)	3 (100)	0.540
System upgrade	10 (3)	4 (3)	3 (3)	3 (6)	0 (0)	0 (0)	0 (0)	0.800
Procedure time (h)	1.8 ± 0.9	1.8 ± 0.7	1.9 ± 1.1	2.1 ± 1.2	1.9 ± 0.7	1.7 ± 0.7	2.3 ± 0.9	0.482

Values are given as the *n* (%) or mean ± standard deviation

*AT* antithrombotic therapy, *CIED* cardiac implantable electronic device, *CRT-D* cardiac resynchronization therapy-defibrillator, *CRT-P* cardiac resynchronization therapy-pacemaker, *DAPT* dual antiplatelet therapy, *ICD* implantable cardioverter-defibrillator, *OAT* oral anticoagulant therapy, *PM* pacemaker, *SAPT* single antiplatelet therapy, *TAT* triple antithrombotic therapy

### Bleeding and thromboembolic risk data of No-AT, OAT, SAPT, OAT + SAPT, DAPT, and TAT groups

The bleeding and thromboembolic risk data of the 6 groups is shown in Table 3. The mean HAS-BLED score, CHADS_2_ score, and CHA_2_DS_2_-VASc score was 1.2, 1.8, and 3.2, respectively. HAS-BLED score ≥3 as a high bleeding risk parameter was 21 (7 %) and significant difference among the 6 groups. CHA2DS2-VASc score ≥2 as a considerable thromboembolic risk parameter was 247 (82 %) and significant difference among the 6 groups.

**Table 3:** Comparison of the bleeding and thromboembolic risk data among No-AT, OAT, SAPT, OAT + SAPT, DAPT, and TAT groups

	Total (*n* = 300)	No-AT (*n* = 129)	OAT (*n* = 89)	SAPT (*n* = 49)	OAT + SAPT (*n* = 20)	DAPT (*n* = 10)	TAT (*n* = 3)	*p* value
Bleeding risk data								
HAS-BLED score	1.2 ± 0.9	0.8 ± 0.7	1.0 ± 0.7	2.0 ± 0.8	2.1 ± 0.6	2.0 ± 1.0	1.7 ± 0.6	<0.001
HAS-BLED score ≥3	21 (7)	2 (2)	3 (3)	10 (20)	2 (10)	4 (40)	0 (0)	0.001
Thromboembolic risk data								
Prior stroke/recurrent venous thromboembolism	34 (11)	4 (3)	14 (16)	8 (16)	7 (35)	0 (0)	1 (33)	<0.001
Active cancer	0 (0)	0 (0)	0 (0)	0 (0)	0 (0)	0 (0)	0 (0)	–
Thrombophilia	0 (0)	0 (0)	0 (0)	0 (0)	0 (0)	0 (0)	0 (0)	–
CHADS_2_ score	1.8 ± 1.3	1.2 ± 1.2	1.9 ± 1.1	2.5 ± 1.3	2.8 ± 1.3	1.8 ± 0.8	2.7 ± 2.1	<0.001
CHA_2_DS_2_-VASc score	3.2 ± 1.8	2.5 ± 1.6	3.3 ± 1.5	4.3 ± 1.7	4.7 ± 1.6	3.4 ± 1.0	4.3 ± 2.5	<0.001
CHA_2_DS_2_-VASc score ≥2	247 (82)	88 (68)	80 (90)	47 (96)	19 (95)	10 (100)	3 (100)	<0.001

Values are given as the *n* (%) or mean ± standard deviation

*AT* antithrombotic therapy, *DAPT* dual antiplatelet therapy, *OAT* oral anticoagulant therapy, *SAPT* single antiplatelet therapy, *TAT* triple antithrombotic therapy

### Perioperative complication data of No-AT, OAT, SAPT, OAT + SAPT, DAPT, and TAT groups

**Fig. 1:**
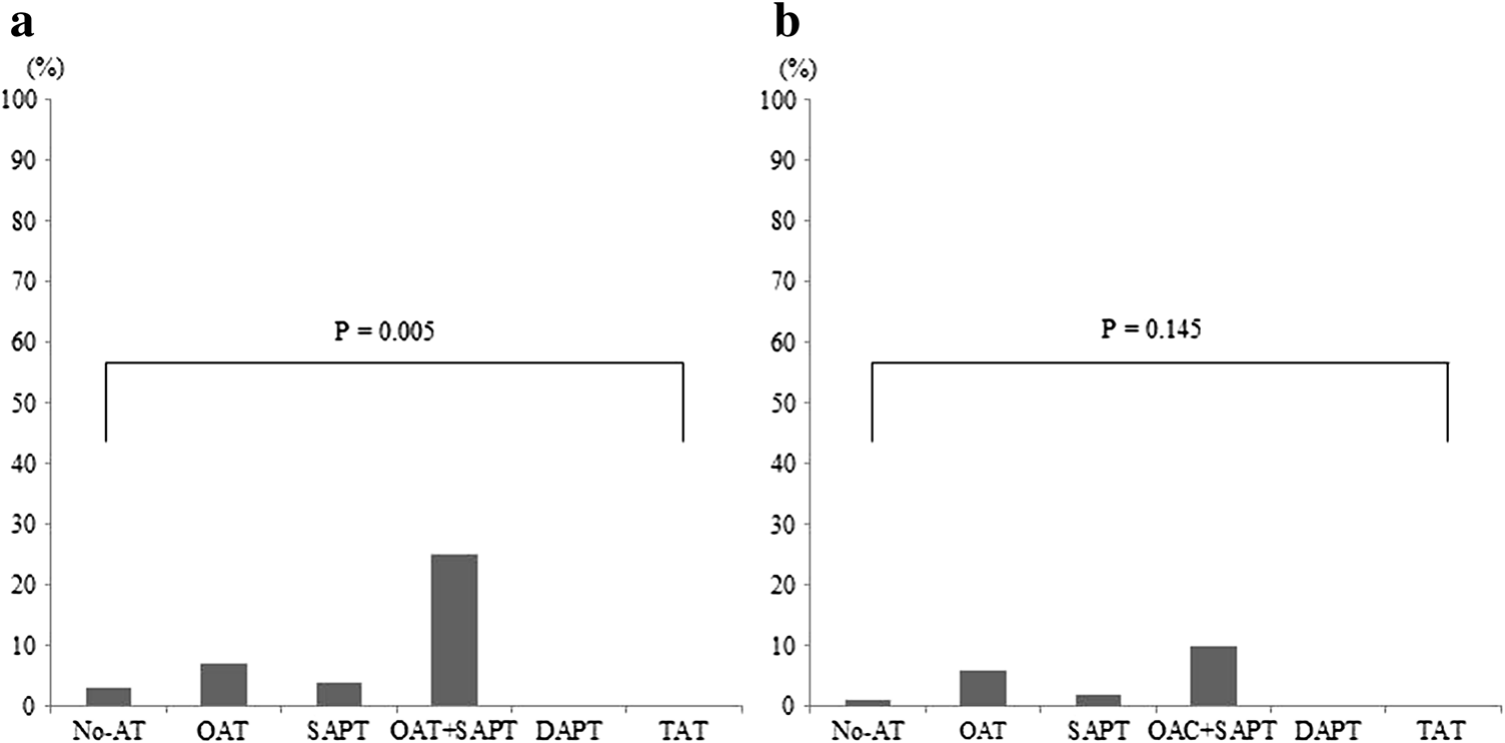
**a** Pocket hematoma and clinically significant pocket hematoma among No-AT, OAT, SAPT, OAT + SAPT, DAPT, and TAT groups. The frequency of PH was 3, 7, 4, 25, 0 and 0 % in No-AT, OAT, SAPT, OAT + SAPT, DAPT and TAT groups, respectively, and was significantly different among the 6 groups. **b** The frequency of clinically significant PH was 1, 6, 2, 10, 0 and 0 % in No-AT, OAT, SAPT, OAT + SAPT, DAPT and TAT groups, respectively, and was not different among the 6 groups. *AT* antithrombotic therapy, *DAPT* dual antiplatelet therapy, *OAT* oral anticoagulant therapy, *PH* pocket hematoma, *SAPT* single antiplatelet therapy, *TAT* triple antithrombotic therapy

PH and clinically significant PH occurred 17 (6 %) and 9 (3 %) patients, respectively. The frequency of PH was significantly different, but the frequency of clinically significant PH was not different among the 6 groups (Fig. 1). The gastrointestinal bleeding occurred 2 (1 %) and there was no significant difference among the 6 groups (*p* = 0.113). No subjects had any cardiac tamponade and cerebral bleeding.

Thromboembolic event occurred in only one patient and there was no significant difference in the frequency of that event among the 6 groups (*p* = 0.795).

### Predictors of clinically significant PH

The frequency of VHD [OR 6.8; 95 % confidence interval (CI) 1.4–27.8; *p* = 0.010] or high HAS-BLED score (OR 2.2; 95 % CI 1.1–4.3; *p* = 0.021) were major predictors of clinically significant PH by univariate analysis, as shown in Table 4. In addition, low body mass index (OR 0.78; 95 % CI 0.6–0.98; *p* = 0.040) was also a significant predictor of clinically significant PH by univariate analysis. The frequency of VHD (OR 7.2; 95 % CI 1.3–35.0; *p* = 0.015) or high HAS-BLED score (OR 2.5; 95 % CI 1.2–5.3; *p* = 0.014) remained significant independent predictors of clinically significant PH developing by multivariate analysis.

**Table 4:** Univariate and multivariate analyses of predictors of clinically significant PH

	Univariate analysis			Multivariate analysis		
	Odds ratio	*p* value	95 % CI	Odds ratio	*p* value	95 % CI
Male gender	2.71	0.219	0.64–18.40			
Age (years)	1.05	0.126	0.99–1.12			
BMI	0.78	0.040	0.61–0.98	0.79	0.076	0.61–1.01
Valvular heart disease	6.77	0.010	1.35–27.79	7.18	0.015	1.31–34.98
Ischemic heart disease	1.31	0.739	0.19–5.62			
CHADS2 score	1.28	0.317	0.77–2.04			
HAS-BLED score	2.20	0.021	1.11–4.33	2.50	0.014	1.20–5.34
High joule device	1.59	0.495	0.41–6.54			
Generator exchange	2.21	0.244	0.57–9.11			
Operation time (h)	1.50	0.140	0.81–2.47			
Blood hemoglobin (g/dl)	0.85	0.387	0.57–1.23			
Serum creatinine (mg/dl)	1.19	0.624	0.42–2.03			

*BMI* body mass index, *CI* confidence interval

## Discussion

To the best of our knowledge, this is the first report on the comprehensive data concerning the safety of continuous multi AT during CIED implantations. The results of this study revealed that continuous multi AT was acceptable but high HAS-BLED and VHD were independent predictors of clinically significant PH during CIED implantations. These findings suggested that continuous multi AT may be tolerated, but patients with high HAS-BLED score or VHD would require a careful attention during CIED implantations.

Although the rate of clinically significant PH did not differ significantly among the 6 groups in our study, 5 % of that complication rate in AT was relatively high. Previous studies revealed that a perioperative continuous single AT was associated with an incidence of PH of 1.9–6.6 % [20–27]. Thus, the clinically significant PH rate of the multi AT in our study may be acceptable.

The incidence of thromboembolic events was rare and did not differ significantly among the 6 groups in this study. The thromboembolic event rate of the single AT was 0–1 % in previous studies [20–23]. Continuous multi AT may be effective in suppressing thromboembolic events within the perioperative period.

High HAS-BLED score and VHD were independent predictors of clinically significant PH during CIED implantations in this study. There is no report revealing that VHD is associated with bleeding complication during CIED implantations; however, one previous study said that the observed risk of bleeding was higher with AT in patients with VHD compared with patients without VHD [28]. Since VHD is a considerable disease, specific attention is needed in this population during CIED implantations.

There were many warfarin users in our study, so our results are acceptable for patients with warfarin. The mean PT-INR of the warfarin users in this study was 1.7 and it was a low control level considering the previous data [29, 30]. The bleeding risk in Asians is greater than that of the people in the other countries [31]. Previous Japanese registries revealed that a PT-INR of 1.6–2.6 was safe and effective in preventing thromboembolic events, particularly in patients aged ≥70 years old [32–34]. The recommended PT-INR in the Japanese guidelines is 2.0–3.0 and 1.6–2.6 for patients aged <70 years and ≥70 years, respectively [33]. Thus, our control level of the PT-INR was acceptable. In contrast, only 1 % of the patients used NOACs, so we could not sufficiently evaluate the safety and efficacy of NOACs. Previous studies revealed that continuous NOAC use was safe during CIED implantations [10, 11]. Thus, multi AT including NOACs may be safe and effective.

## Limitations

There are several limitations to our study. The patient number in this study, especially for NOAC users, was very small. The evaluation of large number in this topic is needed by a future study. Furthermore, procedures performed by experienced operators carry a lower risk of clinically significant PH compared with trainees and less experienced operators [25, 26, 35]. Our hospital is a high-volume center (mean operation procedures, 500 per year) for CIED implantations, and experienced operators performed all surgeries in our study. A future multi-center study is needed to resolve this problem.

## Conclusions

Continuous multi AT may be tolerated, but patients with high HAS-BLED score or VHD would require a careful attention during CIED implantations.

## Abbreviations

ACE: Angiotensin converting enzyme
ANOVA: Analysis of variance
ARB: Angiotensin receptor blocker
AT: Antithrombotic therapy
BMI: Body mass index
CHF: Congestive heart failure
CI: Confidence interval
CIED: Cardiac implantable electronic device
CRT: Cardiac resynchronization therapy
CRT-D: Cardiac resynchronization therapy-defibrillator
CRT-P: Cardiac resynchronization therapy-pacemaker
DAPT: Dual antiplatelet therapy
DM: Diabetes mellitus
HT: Hypertension
ICD: Implantable cardioverter defibrillator
OAT: Oral anticoagulant therapy
NOAC: Novel oral anticoagulant
PH: Pocket hematoma
PM: Pacemaker
PT-INR: Prothrombin time-international ratio
SAPT: Single antiplatelet therapy
SD: Standard deviation
TAT: Triple antithrombotic therapy
TIA: Transient ischemic attack
VHD: Valvular heart disease
